# Rapid Brownian Motion Primes Ultrafast Reconstruction of Intrinsically Disordered Phe-Gly Repeats Inside the Nuclear Pore Complex

**DOI:** 10.1038/srep29991

**Published:** 2016-07-29

**Authors:** R. Moussavi-Baygi, M. R. K. Mofrad

**Affiliations:** 1 Molecular Cell Biomechanics Laboratory, Departments of Bioengineering and Mechanical Engineering, University of California, Berkeley, CA 94720, USA; 2 Molecular Biophysics and Integrative Bioimaging Division, Lawrence Berkeley National Laboratory, Berkeley, CA 94720, USA

## Abstract

Conformational behavior of intrinsically disordered proteins, such as Phe-Gly repeat domains, alters drastically when they are confined in, and tethered to, nan channels. This has challenged our understanding of how they serve to selectively facilitate translocation of nuclear transport receptor (NTR)-bearing macromolecules. Heterogeneous FG-repeats, tethered to the NPC interior, nonuniformly fill the channel in a diameter-dependent manner and adopt a rapid Brownian motion, thereby forming a porous and highly dynamic polymeric meshwork that percolates in radial and axial directions and features two distinguishable zones: a dense hydrophobic rod-like zone located in the center, and a peripheral low-density shell-like zone. The FG-meshwork is locally disrupted upon interacting with NTR-bearing macromolecules, but immediately reconstructs itself between 0.44 μs and 7.0 μs, depending on cargo size and *shape*. This confers a *perpetually-sealed* state to the NPC, and is solely due to *rapid Brownian motion* of FG-repeats, not FG-repeat hydrophobic bonds. Elongated-shaped macromolecules, both in the presence and absence of NTRs, penetrate more readily into the FG-meshwork compared to their globular counterparts of identical volume and surface chemistry, highlighting the importance of the shape effects in nucleocytoplasmic transport. These results can help our understanding of geometrical effects in, and the design of, intelligent and responsive biopolymer-based materials in nanofiltration and artificial nanopores.

Ubiquitously common in all living organisms, intrinsically disordered proteins (IDPs) can be classified as important extensions of the protein kingdom^1^. Under physiological conditions, the structural dynamics, extremely intrinsic flexibility, and the presence of diverse functional motifs along their backbone enable IDPs to rapidly and promiscuously interact with different binding partners^2^. As such, the lack of a folded structure confers a unique and diverse functionality to IDPs featured in a variety of cellular processes including signaling, cycle regulation, transcription, translation, and pathways control^3^. Remarkably, IDPs also exhibit a stimulus-dependent response under different physical and geometrical conditions, opening new doors for exploring them as stimuli-responsive biopolymers. Thus, in recent years, a great deal of interest has been generated in fundamental understanding of their peculiar biophysics^4^.

One of the impressive examples of IDPs functionality that has provided inspiration for biomimetic materials and processes^5^ occurs inside the nuclear pore complex (NPC)^6^, comprising a high concentration of nucleoporins (Nups) that have intrinsically disordered domains rich in phenylalanine-glycine (FG)-repeat motifs^7^. There are a total of 11 different types of FG-Nups per NPC, each of which is present in a multiple of 8, 16, or 32, owing to the high degree of symmetry in the octagonal architecture of the NPC^8^. The intrinsically disordered domains of FG-Nups that are confined in, and tethered to the interior of the NPC channel, form a ‘sticky soup’ of end-tethered, delicate biopolymers dangling inside the cramped space of the channel, rendering the NPC a sorting machinery that selectively facilitates the bidirectional nucleocytoplasmic transport of macromolecules^9^. As such, the heterogeneous mixture of FG-repeats establish a robust permeability barrier inside the NPC channel^10^ that accommodates a tremendous rate of transport of about 1000 translocation events per second per NPC, amounting to over 100 MDa/s/NPC^11^. The permission to pass through the permeability barrier is granted to a macromolecule only if it is bound to a nuclear transport receptor (NTR), which carries hydrophobic binding spots on the surface and possesses a net negative charge^12^.

Despite more than two decades of dedicated research, the mechanistic understanding of such a robust permeability barrier is still under debate. This is mainly due to the lack of comprehensive understanding of how FG-repeats precisely behave, and which conformations they adopt within the NPC channel *in vivo*. The concentrated, heterogeneous mixture of delicate FG-repeats, featuring a large number of hydrophobic and charged residues, densely tethered to and confined in the nanometer-sized concave surface of the NPC channel, have concertedly made it impractical to inspect their precise conformational behavior not only *in vivo,* but even *in vitro* as well. Today, there is no imaging technique capable of visualizing these biopolymers within the NPC channel in their functional state^10^, the lack of which has left plenty of room for speculation on the characteristics and formation of the FG-repeats-based permeability barrier. Strikingly, depending on one’s interpretation of FG-repeats conformation inside the NPC channel, one can propose different models for nucleocytoplasmic transport, as have been proposed in the past two decades (reviewed in ref. 13). It is thus crucial to first develop a clear understanding of the conformational behavior of FG-Nups *per se* without the mass flux bias across the NPC^14^. Although insightful, so far the conformational studies on FG-repeats have mainly focused on the single-type FG-Nup in an unconfined geometry and typically non-tethered state, where the results are extrapolated to the heterogeneous mixture of all FG-Nups, end-tethered to the nanometer-sized confined geometry of the NPC channel^10^. There are other valuable studies on the conformation of heterogeneous mixture of FG-Nups^15^, or the energy landscape within the pore^16^, but they lack dynamical aspects and the Brownian motion of these highly dynamic biopolymers.

Experimentally-driven computational biophysical modeling approaches can contribute toward filling the gap by providing unique microdynamical insights at high spatiotemporal resolutions. At one extreme are all-atom molecular dynamics studies^17^,^18^ that are exceptionally useful in predicting the atomistic details of processes, but are limited to small domains and can span times up to several hundreds of nanoseconds due to computational feasibility. At the other extreme are probabilistic approaches that can span the entire transport cycle of the NPC populations at the price of losing structural details^19^. As a compromise between spatiotemporal resolution and computational feasibility, coarse-grained modeling approaches have emerged recently, where the structural features are elaborated depending on the level of coarse-graining^20^,^21^.

## Results

### FG-repeats form a nonuniform, porous, and highly dynamic meshwork, having a central dense zone with a dynamic hydrophobic core, and percolating in radial and axial directions

**Figure 1:**
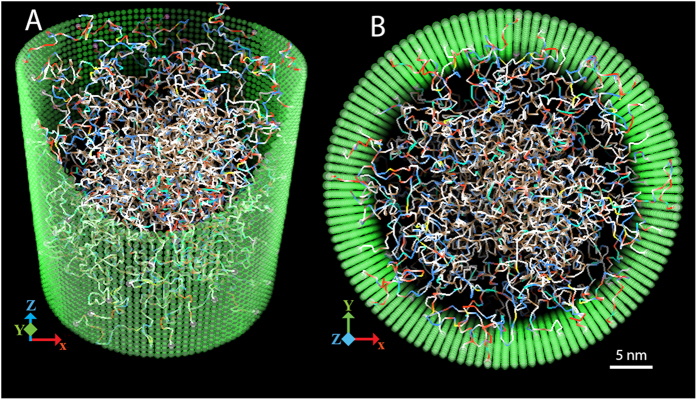
Central channel with all FG-repeats after equilibration. The wall is shown as semi-transparent. Different monomers in FG-domains are colored according to their properties (see SI for details) as following: brown is hydrophobic (HB), yellow is hydrophobic-negative (HN), cyan is hydrophobic-positive (HP), red is purely negative (PN), blue is purely positive (PP), and white is hydrophilic with zero net charge (HL). (**A**) Side view. A part of the wall is cut for a better representation of inside the channel. (**B**) Top view.

Throughout the simulation, an extremely dynamic, cohesive meshwork of unstructured FG-repeats formed inside the channel (Fig. 1), reminiscent of a bowl of ‘oily spaghetti’^22^ that *seemingly* fills the channel. We repeated the simulation 10 times with different initial conditions and verified that the apparent meshwork was repeated, and thus the formation of the meshwork did not depend on the initial conditions. We also demonstrated that the formation of the meshwork was not an artifact caused by the pre-exposed hydrophobic as well as charged residues due to the initially cramped configurations of FG-Nups within the channel (see SI).

**Figure 2:**
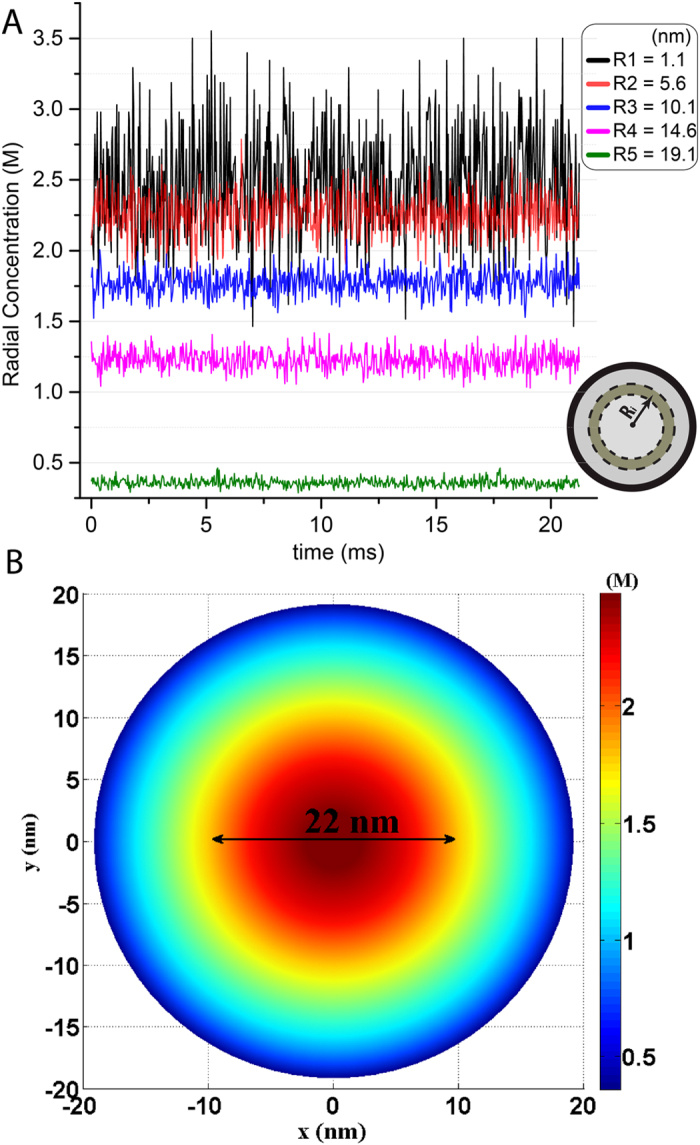
Radial concentration of FG-repeats inside the NPC channel. (**A**) Time-dependent concentration fluctuations in radial direction at five different radii inside the channel. At each *R*_*i*_, the concentration is calculated within a cylindrical shell (the ring inside the yellow circle) with a width of the monomer’s vdW diameter. (**B**) Time-averaged 2D map of radial concentration of all AAs within the FG-repeat domains. The concentrations are averaged along the channel main axis over 20 *ms*. The diameter of the rod-like high-density zone is about 22 nm.

Next, we explored the extremely dynamic characteristics of this FG-meshwork, arising from the rapid Brownian motion of individual FG-repeats. The total concentration of all AAs within the disordered domains of FG-Nups localized to the yeast NPC channel is about 1.2 M. However, due to the highly dynamic nature of unstructured domains, the concentration is significantly fluctuating (Fig. 2A and Movie S1). The time-dependent concentration fluctuations in radial direction shows that the fluctuations are largest in the middle and smallest near the wall (Fig. 2A). This is because of accumulation of free ends along the central axis and more stationary situation near the wall^23^.

The time-averaged 2D map of all AAs’ radial concentration (Fig. 2B) exhibits a 6-fold increase in concentration from near the wall (0.4 M) to the middle (2.4 M). This is a signature of formation of a nonuniform meshwork with a dense zone in the middle that weakens toward the low-density zone near the wall. While the low-density zone resembles a sparse polymeric shell in the wall vicinity, the dense zone features a rod-like high density region that is about 22-*nm* thick and extends axially along the channel (Figs 2B and 7).

Notably, the average density of the central zone decreases as the diameter of the channel increases, in agreement with the behavior of single-type FG-Nups grafted inside the solid-state nanopores^24^. At higher diameters of the pore, the central zone disappears, and instead, the radial concentration spreads more evenly within the channel (see Fig. S5). This suggests that the channel diameter affects the formation of different zones within the FG-meshwork confined to the channel interior. However, in the current study we consider the channel diameter of the yeast NPC that is 40 nm^25^.

To further analyze the rod-like high density zone we determined its constituent residues and the type of dominant interactions therein. The time-averaged 2D map of the single-type AA radial concentration shows a relatively large number of Phe—the most hydrophobic residue^26^—in the rod-like zone with a concentration of about 1 M (Fig. S6). This suggests that the most hydrophobic region of the FG-meshwork is somewhere along the rod-like zone.

**Figure 3:**
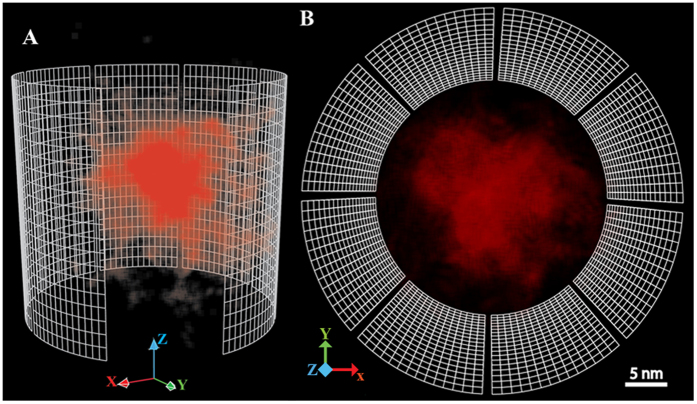
The 3D spatial density map of inter- and intra-FG-repeats hydrophobic interactions inside the channel. We pinpointed every single hydrophobic interaction and recorded its coordinates along with the strength of interaction throughout the simulation. Each dim point shows a single hydrophobic interaction, and thus, the color intensity is proportional to the number of hydrophobic interactions. The channel is represented as a wired frame for clarity. (**A**) side view and (**B**) top view snapshots.

Nevertheless, since the concentration map is a 2D radial projection, it does not convey information about dynamics and the accurate location of hydrophobic interactions. Thus, we dissected the time-evolution of hydrophobic interactions within the FG-meshwork through the time-dependent 3D spatial density map. The 3D map of hydrophobic interactions affirmed that the majority of those interactions occur in the central part, both radially and axially, forming a dynamic hydrophobic core (Fig. 3 and Movies S2 and S3).

We then analyzed the connectivity profile of the meshwork, using the geometrical percolation theory^27^ as adopted in polymer physics^28^. Using this powerful mathematical tool, we determined that the FG-meshwork percolates inside the channel both in radial and axial directions (Fig. S7), implying that the FG-meshwork exhibits a geometrical connectivity, similar to a gel. This further implies the physiological concentration of FG-repeats is sufficient to form a connecting meshwork inside the NPC, but does not necessarily denote that the channel is *uniformly* filled up as implied by the hydrogel model^29^.

Next, we examined the porosity of the FG-meshwork using the monomer-monomer pair distribution function (PDF)^30^, with the pair of monomers belonging to two distinct FG-Nups. The location of the first peak in the PDF plot represents the most probable pairwise distance between monomers^30^, and thus is a measure of the meshwork porosity. We found that the dominant size of passive pores within the FG-meshwork is about 6 *nm* (Fig. S8-A), setting an upper limit to the free diffusion of small particles across the NPC. Yet, because the meshwork is spatially heterogeneous, we performed the *zonal* PDF within the high- and low-density zones separately, and determined that the peak location shifts to 4 *nm* in the high-density zone (Fig. S8-B), implying the porosity inside the rod-like zone is tighter compared to the entire meshwork. The PDF plot for the low-density zone, however, is very similar to the entire FG-meshwork (Fig. S8-C), suggesting that the porosity of the entire FG-meshwork is mainly dictated by this zone.

### The FG-meshwork locally collapses upon interacting with the NTR-bearing macromolecule, but autonomously reconstructs itself with a characteristic time between sub-microsecond and ≤7.91 *μs*, depending on the macromolecular size and shape

For efficient nucleocytoplasmic transport cycles, the NPC channel must constantly accommodate macromolecules of different sizes. If the channel is sealed by a dense FG-meshwork, then the challenging questions would be: Once a NTR-bearing macromolecule perforates and passes through, how fast does the FG-meshwork reconstruct afterward? What is the timescale of reconstruction process in comparison with the milliseconds-long transport time? Does not the constant flow of macromolecules and/or NTR leave the channel in an unsealed state and destroy the permeability barrier?

To explore these questions, we simulated the globular and elongated NTR-bearing macromolecules by placing the macromolecule at the NPC cytoplasmic entry, with the NTR hydrophobic binding spots closely faced against the FG-repeats (see SI for details). The macromolecule was then nudged toward the channel by a small force, on the order of magnitude of the thermal noise (see Table 1). The nudging force was applied only to save computational time by guiding the macromolecule to the right direction and gracefully steering it across the FG-meshwork, instead of wandering around and after a long time finding the correct position. Thus, the force does not have a direct physiological equivalence. Yet, it is too small that does not obscure the native transport of the macromolecule.

Once the NTR-bearing macromolecule reached the mid-channel, it was kept fixed there for 10 *μs* so that the FG-repeats biopolymers around the cargo surface were equilibrated. Then, the cargo was abruptly removed, leaving a cavity inside the meshwork (Movie S4–S7). Subsequently, we measured the time it took for the FG-meshwork to refill the cavity and adopt its pre-cavity conformation—the reconstruction process.

**Figure 4:**
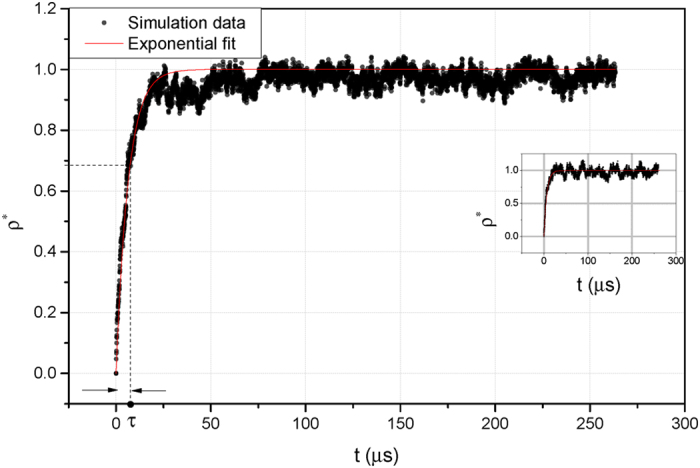
Reconstruction pattern of the FG-meshwork after a 20-nm globular macromolecule passes through. Black dots are simulation data and red line shows the fitted eq. (1). Inset: the reconstruction pattern of the FG-meshwork for an equivalent elongated macromolecule (with the diameter of 11.6 nm and the length of 40 nm).

It appears that the reconstruction process follows a saturating pattern (Fig. 4), for which we suggest the following equation:


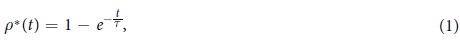


where ρ^*^(*t*) is the time-dependent dimensionless density inside the cavity and equals 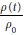, with *ρ*(*t*) being the time-dependent local density and *ρ*_0_ the pre-cavity value of the local density. More importantly,τ, the characteristic time of reconstruction, is the time for the cavity to regain about 2/3 of its initial density, *ρ*_0_.

**Figure 5:**
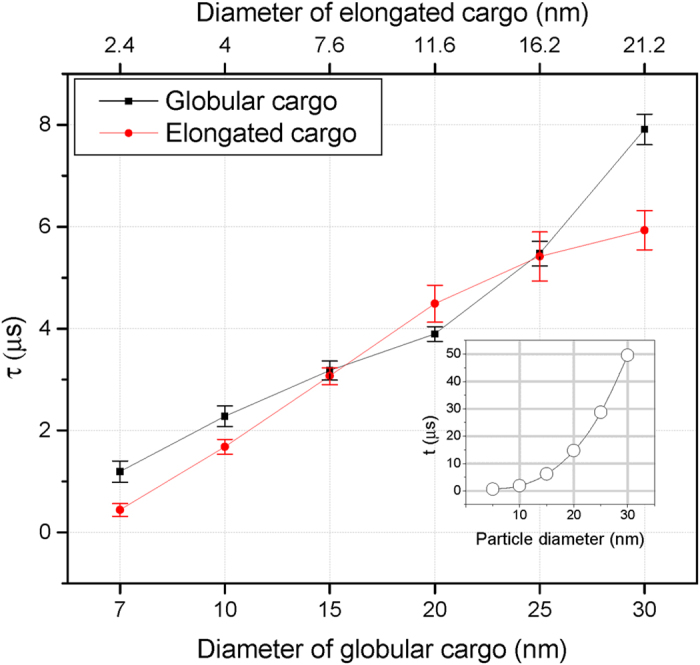
Distribution of the reconstruction characteristic time for different the cavity size of globular and elongated NTR-bearing macromolecules. Inset: The time of local diffusional motion versus the particle diameter for a globular particles diffusing in cytoplasmic viscosity.

Significantly, the reconstruction process follows the similar patterns for both the globular and the elongated macromolecules; initially it proceeds very rapidly and then gradually reaches a saturating phase (Fig. 4). The characteristic time τ is the determining factor for the speed of reconstruction. To find out how τ varies with the cavity size, we studied different sizes of NTR-bearing macromolecules (Fig. 5 and Table 1). Furthermore, to draw conclusion about the shape effects, volumes and surface characteristics of globular and elongated macromolecules were kept the same in a pairwise manner, so that for every globular macromolecule, an elongated counterpart of equal volume and similar surface characteristics was studied (Table 1). For each macromolecule with a particular size and shape, we performed 30 independent simulations, from which the mean of τ was computed.

Generally, τ increases with the cavity size (Fig. 5), indicating there is a direct relation between them. For different sizes of macromolecules, both globular and elongated, we determined that τ varies between half a microsecond and 7.91 *μs* (Fig. 5 and Table 1). Particularly, for globular cargoes beyond 20 *nm*, *τ* increases more rapidly, i.e., relatively it takes longer for the FG-meshwork to reconstruct. This is conceivably due to extensive conformational changes in the FG-meshwork as a result of the large cavity size ≥20 *nm*.

Notably, the reconstruction process here resembles the ‘self-healing’ feature proposed in other studies. The self-healing has been attributed either to inter-FG hydrophobic bonds, or to the continuous presence of the NTR population^31^,^32^. We thus sought to find the underlying root of the autonomous reconstruction and to identify the potential role of hydrophobic interactions in reconstruction by mutating all of the Phe residues to polar residues with the same vdW dimensions. Subsequently, after equilibration of the mutated meshwork we followed the same steps as in the native meshwork in that the macromolecule was forced into and kept fixed in the mid-channel for 10 *μs,* followed by an abrupt removal. We found that the reconstruction process repeated in the mutated meshwork, following the same exponential pattern (Fig. S9), but with a smaller τ, i.e., reconstruction occurs faster than in the native FG-meshwork, diminishing the possible role of FG-FG hydrophobic interactions in the reconstruction process. For instance, for a globular cavity of 20-nm in the mutated meshwork, *τ* = 1.38 ± 0.25 *μs*; this is 65% faster than that in the native FG-meshwork (Fig. S8, and Table 1).

**Figure 6:**
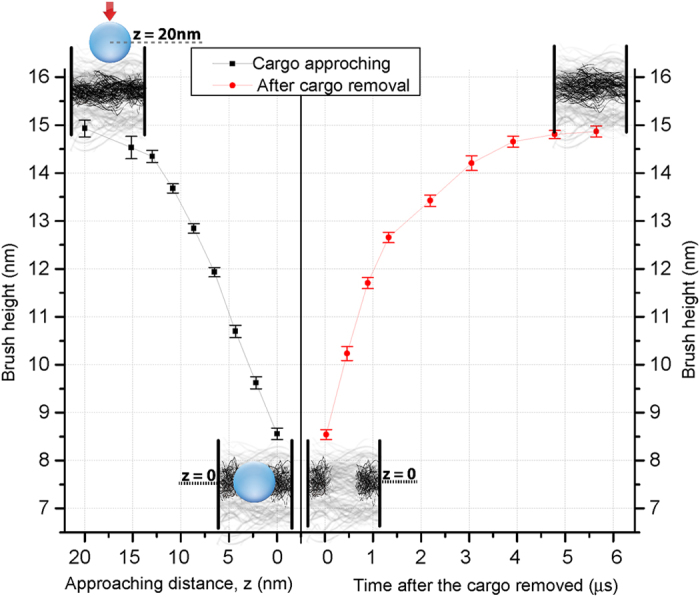
The rapid and reversible collapse of individual FG-repeats in the mid-channel as the NTR-bearing macromolecule approaches from the top. Left graph shows the brush height as a function of approaching distance. Right graph represents after the macromolecule is removed how fast FG-repeats regain their heights as a function of time.

We then looked into how individual FG-repeats conformationally behave upon interacting with the NTR-bearing macromolecule^33^. To this end, we picked a ring of 15-*nm* width in the middle of the channel and monitored the average height of the brush created by the FG-repeats tethered to the ring. As the NTR-bearing macromolecule approaches the ring, FG-repeats show a continuous decrease in their heights, driving them to adopt a collapsed conformation (Fig. 6). As soon as the macromolecule disappears, FG-repeats start regaining their heights, indicating the collapse is promptly reversible.

## Discussion

### Dynamicity underlies concentration fluctuations, facilitating the NTR-bearing macromolecule transport

We observed that the end-tethered FG-repeats formed a porous and extremely dynamic meshwork that constantly jiggles and wiggles, showing high degree of concentration fluctuations inside the NPC channel (Fig. 2A and Movies S1, S4–S8). Indeed, highly dynamic nature of free IDPs and their erratic motions on time scales of sub-nanoseconds is reported^34^. Here, our model suggests that IDPs retain their highly dynamic characteristics, even when they live in a dense cohesive meshwork and are tethered to, and confined in, a compact geometry.

The rapid Brownian motion, and thus the concentration fluctuations within the FG-meshwork, are conceivably among the major underlying factors in efficiently conducting transport. This is achieved by augmenting the back-and-forth motions of the NTR-bearing macromolecule that is engaged in the FG-meshwork through hydrophobic, and to a lesser extent, electrostatic interactions (see following). Analogously, but in a different context, it has been suggested that concentration fluctuations of the actin filaments drive lamellipodia protrusion and retraction, which in turn provokes the cell crawling mechanism^35^.

The concentration fluctuations within the FG-meshwork are reminiscent of the cyclic motions of self-oscillating gels induced by an oscillatory chemical reaction called the Belousov-Zhabotinsky (BZ) reaction^36^. In those systems, periodic chemical energy of the BZ reaction is converted to mechanical oscillations within the polymeric meshwork. Indeed, fluctuations in self-oscillating hydrogels has been harnessed for mass transport and cargo delivery purposes^37^,^38^. However, there is a fundamental difference between those systems and the NPC in that no chemical reaction occurs inside the FG-meshwork, nor is there any external source of energy to wriggle the FG-meshwork. Instead, we propose that the thermal noise, spreading through the geometrically confined NPC channel that hosts numerous transient, individually weak hydrophobic and electrostatic bonds, along with the delicate structures of the *end-tethered* FG-repeats, *en masse* produce incessant rapid Brownian motion, leading to continuous concentration fluctuations in the NPC channel.

### Ultrafast reconstruction of the FG-meshwork arises solely from rapid Brownian motion of FG-repeats, keeping the NPC channel in a perpetually-sealed state

We quantified the reconstruction pattern and time for the FG-meshwork in detail and proposed a time-dependent relation for this process (Eq. 1) which is biphasic with a rapid and a slow phase (Fig. 4). The reconstruction occurs mainly during the first phase, which is mainly entropically driven; a cavity within a dense meshwork is entropically highly unfavorable, and thus, once the macromolecule passes through, the meshwork quickly rearranges itself. Once the configurational entropic cost is compensated, the density fluctuations within the cavity, *ρ*(*t*), continues more slowly with lots of ‘small-amplitude’ peaks and valleys during the saturating phase (Fig. 4). Imaginably, favorable inter- and intra-FG-meshwork hydrophobic as well as electrostatic interactions play a more visible role in this phase.

Our results revealed that the characteristic time of reconstruction, *τ*, lies anywhere between 0.44 ± 0.12 *μs* and 7.91 ± 0.30 *μs*, depending on the macromolecule size and shape (Fig. 5 and Table 1). Given the transport time of a single macromolecule is anywhere between 3 *ms* to several seconds^20^,^21^,^39^,^40^,^41^,^42^,^43^,^44^,^45^,^46^, the reconstruction occurs three to seven orders of magnitude faster than the actual transport. This indicates that the reconstruction process is ultrafast and ‘instantaneous’ compared to the timescale of the entire transport process.

More precisely, τ is even shorter than the time of *local* diffusional motion of the macromolecule. Here, by *local* we mean the time it takes for a particle to diffuse a distance equal to its characteristic length, *L*. For example, for a globular cargo of 20 *nm* in diameter it takes about <*t*> = <*L*^2^>/6*D* ≅ 15.0 *μs* to diffuse its diameter in cytoplasmic viscosity^47^. Since the cargo diffusion inside the NPC channel is slower than that within the cytoplasm^45^, 15.0 *μs* is the lower bound on the *local* diffusional time of a 20-*nm* globular cargo inside the NPC. Yet, τ for the cavity created by the same cargo is 3.89 ± 0.14 *μs* (±*SEM*) (Table 1), meaning that the reconstruction process is at least fourfold faster than the *local* diffusional motion. This implies that the cargo does not leave any void behind itself, suggesting the NPC channel is perpetually sealed so that the traffic of macromolecules does not lead to breaking the permeability barrier or ‘leaking’.

The key factor here is the existence of different timescales involved in the game: the FG-meshwork reconstruction occurs three to seven orders of magnitude faster than the transport process. Moreover, the reconstruction characteristic time is at most 1/4 of the cargo *local* diffusional time, i.e. the reconstruction is accomplished while the macromolecule is still around. Consistently, based on the difference in influxes of nonspecific (inert) versus NTR-bearing cargoes through an *in vitro* hydrogel obtained from saturated FG/FxFG repeats of the Nsp1, it was shown that the ‘lesions’ created in the hydrogel were short-lived, and thus, the hydrogel ‘immediately’ resealed behind particle translocations^31^. Nonetheless, to the best of our knowledge, the mechanistic details of reconstruction have never been investigated thoroughly, regardless of which conformational behavior FG-repeats adopt.

We also showed here this ultrafast reconstruction, reminiscent of ‘self-healing’, is merely due to the highly dynamic characteristics of the FG-meshwork, and not due to the inter- or intra-FG-Nup hydrophobic interactions, nor the presence of NTRs. In a study by Görlich and co-worker, based on a single-type FG-Nup (Nup49p or Nup57p) synthetic hydrogel at macroscopic scale *in vitro*, it was suggested that the ‘self-healing’ stems from inter-FG-repeats hydrophobic cross-linking^31^. More recently, however, from the basis of another single-type FG-Nup (hNup62) grafted on a plane (unconfined) geometry, Lim and co-workers argued that the ‘self-healing’ was due to the continuous presence of kap β population and was triggered by the kap β-FG-Nup binding avidity^32^.

Obviously, in the current study neither NTR nor NTR-bearing macromolecules are continuously present inside the NPC channel. Particularly, after the NTR-bearing macromolecule is abruptly removed, it is solely the FG-meshwork on its own that autonomously fills the cavity and reconstructs itself with no aid from the NTR or NTR-bearing cargo. Therefore, our model does not support the notion that the ‘self-healing’ is conferred by the NTR or any other external agent as proposed by Schoch *et al.*^32^. Likewise, the ‘self-healing’ character does not stem from inter- or intra-FG-repeats hydrophobic interactions, as the mutated meshwork, lacking hydrophobic interactions, reconstructs itself even faster than the native FG-meshwork (Fig. S9). For example, for a 20-*nm* globular cavity in the mutated meshwork, τ is 1.38 ± 0.25 *μs* (±*SEM*) (Fig. S9); about three times smaller than that in the native FG-meshwork (Table 1). Indeed, this is because the mutated meshwork is non-cohesive and lacks the ‘stickiness’, and thus, entropic repulsions are not counterbalanced by favorable hydrophobic interactions. Incidentally, this also explains why the amplitudes of peaks and valleys during the second phase of reconstruction in the mutated meshwork are larger compared to the native FG-meshwork (compare Figs 4 and S9). Therefore, the molecular basis of the reconstruction process is not due to the inter- or intra-FG-repeats hydrophobic interactions. Instead, it solely arises from the rapid Brownian motion of the confined, end-tethered FG-repeats, manifested in the dynamicity of the FG-meshwork.

Although we do not capture mechanical characteristics of the FG-meshwork in the current study, it has been shown that the Phe → Ser mutation in the Nsp1 synthetic hydrogel changes mechanical properties and converts the semi-solid gel into a more fluidic aggregation^48^. However, a generalization of this observation to the interior of the NPC channel is questionable, as four major contributing factors in determining the FG-meshwork conformation were absent in that study. These factors are the FG-meshwork heterogeneity, end-tethering, confinement, and nano-scale size effects of the channel.

Our model also suggests that *individual* FG-repeats undergo *rapid* collapse upon interacting with NTR-bearing macromolecules (Fig. 6). The collapse is rapidly reversible, i.e., the moment the NTR-bearing macromolecule dissociated from the FG-Nup, disordered domains stretch away from the wall rapidly due to the entropic restoring force. Indeed, plane-grafted disordered domains of Nup153 was shown to undergo a reversible and rapid transition from polymer brush to collapsed state when they interact with kap β^33^. Here, however, we extend that result to the interior of the NPC, with the heterogeneous mixture of all FG-Nups confined to and grafted in the channel. This agrees with a recent theoretical and computational study where the self-consistent field theory as portrayed by the strong-stretching theory was employed to investigate a polymer-brush-based nanovalve^49^. Our model suggests that the *rapid* collapse of individual FG-domains is truly an essential step for nucleocytoplasmic transport, because in its absence the NTR-bearing macromolecule cannot quickly and efficiently penetrate into the FG-meshwork (see following).

### Within the yeast NPC channel, the FG-meshwork is composed of two porous zones: a central high-density rod-like zone, and a peripheral low-density shell-like zone

**Figure 7:**
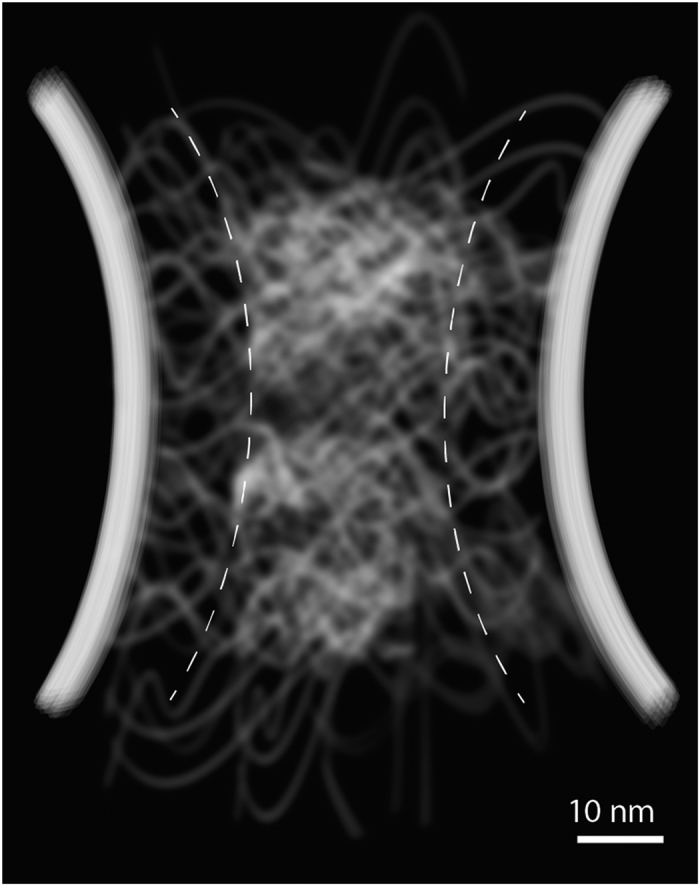
The side-view of the yeast NPC central channel based on the prediction of the current study, representing two zones inside the FG-meshwork (Fig. 2). Dashed lines are to guide the eye to distinguish between two zones.

Our results markedly show that there are two distinguishable regions inside the channel: a rod-like high-density central zone and a polymeric shell-like low-density peripheral zone (Fig. 7). The dense zone is largely hydrophobic with a diameter of about 22 *nm,* having the highest concentration of Phe residues (Fig. 2B and S6) that extends along the channel axis and possesses a dynamic hydrophobic core (Fig. 2B and Movie S2 and S3). This hydrophobic core is reversibly dissolved upon interaction with NTR-bearing macromolecules (Fig. 6 and Movies S4-S7). The low-density zone is about 9 *nm* thick next to the channel wall and resembles a polymeric shell (Fig. 2B).

In the study of Yamada *et al.*, based on the stoichiometry, topology, hydrodynamic dimensions, and *speculated* interactivity of the yeast FG-Nups, the existence of two zones of traffic in the channel was proposed^15^. The low-density zone we observed here closely resembles what they termed ‘zone 2’ in the vicinity of the channel wall. Their proposed ‘zone 1’ located in the middle of the channel has a hydrophobic environment, consistent with the hydrophobic rod-like high density zone in our model. However, the zone 1 in Yamada *et al.*’s study features hollowness, as opposed to the rod-like zone observed here, which is densely packed with hydrophobic residues. This is believably because Yamada *et al.*’s study did not consider the dynamics, geometrical restrictions, and the end-tethering of FG-repeats—three important elements that literally underpin every aspect of the conformational behavior^14^. Indeed, the existence of a dense region in the central part of the channel has been also suggested by a recent high-resolution structural study in the human NPC.

Remarkably, the presence of a cylindrical ‘central plug’ with low electron density, or a ‘central transporter’ with a weak smeared density in the channel was first proposed more than two decades ago by several independent studies in the *Xenopus* and yeast NPCs^50^,^51^. Based on these studies, the central plug was hypothesized to adopt two distinct states, “open” and “closed”^50^. It had also been realized that the central plug was basically the same as transporter^50^. However, later on, it was presumed that the central plug was, at least in part, the remnants of the collapsed nuclear basket^52^, or the cargo caught in transit during preparation^53^. Importantly, some later studies hinted that the central plug might have been the result of aggregation and interaction of FG-repeats inside the channel^15^. Consistently, the spatial arrangement of Phe within FG-Nups in the absence of any dynamics, suggests that Phe residues tend to aggregate mainly toward the central axis of the channel (Fig. S2), implying the static picture supports the formation of a highly hydrophobic region within the middle of the FG-meshwork, in agreement with dynamical observations of our model.

Both zones in our model show porosity with the primary pore size of 4 *nm* in the rod-like dense zone and 6 *nm* in the low-density zone (Fig. S8). The pores can be viewed as passive holes to conduct free diffusions and thus set an upper limit on the size of small particles freely crossing the NPC. It should be noted, however, that these passive holes are not static; rather they constantly form and re-form within different locations of the highly dynamic FG-meshwork.

The passive pore sizes observed in our model closely match those from other qualitative studies reporting the largest size of freely diffusing particles across the NPC^54^. In a detailed study by Mohr and coworkers^55^, authors found the apparent widths of the passive holes were 5.2 *nm*. Indeed, in that study they did not have control over specifying a region inside the NPC to differentiate between zones. Our results benefitted from a more spatial accuracy since we can discriminate between different regions within the NPC and quantify the porosity in each zone. Thus, 5.2 *nm* passive pores^55^ can be interpreted as an average value of the passive holes in two different zones.

### The cargo shape effects

FG-meshwork generally exhibits a mildly faster reconstruction for an elongated cavity than a globular cavity with the same volume (Fig. 5 and Table 1), suggesting elongated cargoes tend to maintain the sealing state of the FG-meshwork more efficiently than their globular counterparts. Notably, compared to a globular-shaped macromolecule, an elongated counterpart also requires about 35% fewer number of hydrophobic binding spots, and about 50% smaller nudging force to penetrate into the FG-meshwork (Table 1), implying that the cargo shape matters in nucleocytoplasmic transport. To further investigate the cargo shape effects, we also examined nonspecific (inert) globular and elongated macromolecules and applied different values of constant nudging forces to find the minimum force required to break the FG-meshwork and overcome the permeability barrier. For the globular-shaped inert cargo of 20 *nm*, the minimum force was approximately 70 *pN*, while for the elongated shape of equal volume, it was 40 *pN*; i.e. about 42% smaller force. Therefore, both for NTR-bearing and inert cargoes, elongated shapes penetrate more readily into the FG-meshwork and thus, cross the pore more efficiently than a globular cargo of equal volume.

This underlines the significance of the cargo shape effects in nucleocytoplasmic transport; particularly, in transport processes where pressure is utilized to send large nonspecific cargoes into the nucleus. In those processes, the translocation of an elongated shape costs less entoropically, and therefore is more favorable. Among others, this might be why the nature has conserved the pressure-driven viral DNA ejection mechanism. Indeed, it has been recently shown that upon docking to the NPC cytoplasmic entry, herpes simplex virus type 1 (HSV-1) ejects its genome to the nucleus using the internal pressure within its capsid that is about 18 *atm*^56^. Intriguingly, this pressure amounts to a force of about 54 *pN* on the HSV-1 highly packed genome, having a nearly cylindrical shape with a diameter of about 20 nm^56^.

While the effects of size have been extensively studied in inter- and intracellular transport in a broad sense^57^, and also more specifically in nucleocytoplasmic transport^20^,^21^,^58^, the shape effects just more recently proved important in cellular uptake and target specificity^57^,^59^,^60^. However, effects of the shape in nucleocytoplasmic transport have been mainly overlooked. Here, our findings imply that shape effects are important in nucleocytoplasmic transport; however, this awaits further in-depth elucidation.

### The hydrophobic affinity difference acts like a stimulus that rapidly ‘opens’ the FG-meshwork and paves the way for efficient transport of NTR-bearing macromolecules

A NTR-bound macromolecule can easily perforates the FG-meshwork with a minimal nudging force of 4.0 *pN* (see Table 1), i.e. in the same order of magnitude as thermal kicks. The quick perforation of the FG-meshwork can be attributed to higher hydrophobic affinity between NTR and FG-repeats than between two FG-repeats (see SI)^54^. Having higher affinity for the NTR, individual FG-repeats would favor to interact with hydrophobic patches on the NTR surface over interacting with themselves once a NTR is around. This would prime the FG-repeats to locally collapse and thus, the cargo can easily penetrate into the FG-meshwork. This is also the principal factor contributing to the rapid collapse of individual FG-repeats upon interacting with a NTR (Fig. 6). In this picture, the ‘affinity difference’ functions as a stimulus that triggers the open state of the FG-meshwork. Indeed, our model suggests that in the absence of such a stimulus there would be a much higher entropic barrier for the NTR-bearing macromolecule to enter the channel. In addition, we found that for a successful transport, the number of sufficient hydrophobic binding spots on the macromolecule surface is proportional to its size (see SI), implying larger cargoes need a proportionally larger number of NTRs to overcome permeability barrier (Table 1) in agreement with previous reports^61^.

Curiously, even after adding net negative charges to the nonspecific cargo surface, the cargo is still not capable of perforating the positively charged FG-meshwork. To break the FG-meshwork and penetrate into, the negatively-charged nonspecific cargo requires the same amount of nudging force that is needed in the absence of surface charge. This highlights the dominating role of hydrophobicity versus charge in crossing the NPC channel, in agreement with a recent study^44^. Therefore, our model corroborates the observation that energetically favorable electrostatic interactions between NTRs and FG-repeats only marginally facilitate the transport process^44^.

## Conclusion

Several lines of evidence support the formation of a hydrogel *in vitro* with permeability properties similar to the NPC^29^,^31^,^48^,^62^,^63^, reinforcing the selective phase/hydrogel model^11^,^61^. The observed hydrogel, however, is homogeneous and produced at macroscopic scale using single-type FG-Nups, where the FG-repeats are not confined to a nanometer-sized geometry, nor are they tethered to an interface. The main question is whether the proposed gelation occurs *inside* the confined NPC channel with the heterogeneous mixture of all types of FG-Nups *tethered* therein. More importantly, the efficiency and speed of ‘resealing’ of such a hydrogel *inside* the NPC, apparently, would mount a formidable challenge to permeability barrier and efficient nucleocytoplasmic transport. The FG-meshwork found in the current study confirms the formation of a percolating meshwork inside the NPC channel, and thus, satisfies the geometrical condition of a gel^27^. Furthermore, we showed that the ‘resealing’ speed, i.e. reconstruction time, of the FG-meshwork arises solely from the ultrafast Brownian motion of FG-repeat biopolymers and is several orders of magnitude faster than transport time. Thus, it is not a limiting factor for the NPC to efficiently conduct the nucleocytoplasmic transport and block nonspecific cargoes. Therefore, the overall picture outlined here is more consistent with a portrait of the NPC where the channel is filled by a hydrogel^31^,^48^,^61^,^62^ rather than being an uncrowded avenue^25^,^64^. Nonetheless, from the viewpoint of polymer physics criteria, whether the FG-meshwork is truly a ‘gel’, or an entangled meshwork of polymers, or a confined polymer brush, or something else, awaits further in-depth mechanical investigation.

More importantly, the current study also suggests that the aggregation of FG-repeats within the NPC channel can be viewed as a novel biopolymeric stimulus-responsive network that immediately changes its conformation in a stimulus-dependent manner. In a shape- and size-dependent way, FG-meshwork ‘rapidly opens up’ in response to hydrophobic affinity difference, while the intrinsic ultrafast Brownian motion of FG-repeat biopolymers ‘quickly closes’ the meshwork as soon as such a stimulus disappears. These call for new investigations on the NPC from novel biopolymer physics viewpoints, combined with shape effects.

## Materials and Method

The current study is a comprehensive extension of our previous 2D works, whose methodology has been stablished and validated^20^,^21^. In brief, here we use Brownian dynamics to develop a 3D biopolymeric model of the NPC central channel^25^,^51^, with all of the FG-Nups that are known to be localized to the channel^15^,^65^. The model enables us to scrutinize conformational behavior of FG-repeats *per se* before, during, and after nucleocytoplasmic transport, and to visualize their native functional states. The yeast NPC central pore was modeled as a rigid cylindrical channel, having a diameter of 40 nm^25^,^51^, with disordered domains of FG-Nups^15^ grafted on the interior of the channel according to their approximate tethering points^65^. The discrete wormlike chain (dWLC) with appropriate choice of discretization level (see SI) was used to model the disordered domains of FG-Nups^20^,^21^, where each monomer composed of five successive amino-acids. The entire systems is placed in an aqueous bath with cytoplasmic viscosity and ionic strength at physiological pH = 7.3 and the body temperature of 37 C. The integrated form of the coupled Langevin equations of motion^66^ are then solved explicitly in time for all monomers: **r**_i_(*t* + Δ*t*) = **r**_*i*_(*t*) + (*D*_*i*_/*k*_*B*_*T*)**F**_*i*_(*t*)Δ*t* + **Λ**_*i*_, where *D*_*i*_ is the diffusion coefficient for the monomer *i*, and **Λ**_*I*_ is the random displacement (see SI for details). Because of the low volume fraction and high aspect ratio of FG-repeats filaments (long and thin), explicit hydrodynamic interactions (HI) between filaments play little role in the confined channel, and can be safely ignored^67^,^68^.

The **F**_*i*_(*t*) is the total conservative force on the monomer *i*, and is composed of the following force-fields: 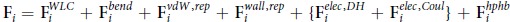. The first two terms are bonded forces that are related to the stiffness and the bending rigidity of the disordered domain (see SI). The third term is the monomer-monomer vdW repulsion, and the fourth term is the channel wall-monomer repulsion. The curly bracket represents the electrostatic force between charged monomers, which is calculated according to the either Debye-Hückel approximation or the pure Coulombic interaction, depending on the charge-charge separation distance. The last term is the entropically-driven hydrophobic attraction between two hydrophobic monomers (see SI for details). Transporting macromolecules are modeled as globular or elongated rigid cargo with the net negative surface charge^69^, bearing adequate number of NTRs on one side of their surface (see SI for details).

## Additional Information

**How to cite this article**: Moussavi-Baygi, R. and Mofrad, M. R. K. Rapid Brownian Motion Primes Ultrafast Reconstruction of Intrinsically Disordered Phe-Gly-Repeats Inside the Nuclear Pore Complex. *Sci. Rep.* **6**, 29991; doi: 10.1038/srep29991 (2016).

## Supplementary Material

Supplementary Information

Supplementary Movie S1

Supplementary Movie S2

Supplementary Movie S3

Supplementary Movie S4

Supplementary Movie S5

Supplementary Movie S6

Supplementary Movie S7

Supplementary Movie S8

## Figures and Tables

**Table 1:** The characteristic times along with the properties of different NTR-bearing globular and elongated macromolecules used in the model.

Globular macromolecule				Elongated macromolecule				
Diameter (nm)	τ (μs) ± SE	Number of hydrophobic binding spots	Nudging force (pN)	Diameter (nm)	Aspect ratio (h/D)	*τ* (μs) ± SE	Number of hydrophobic binding spots	Nudging force (pN)
7	1.20 ± 0.21	10	0.5	2.4	20.83	0.44 ± 0.12	7	0.25
10	2.28 ± 0.20	22	1.0	4.0	12.5	1.68 ± 0.15	15	0.5
15	3.18 ± 0.19	51	2.25	7.6	6.58	3.07 ± 0.17	34	1.25
20	3.89 ± 0.14	91	4.0	11.6	4.31	4.49 ± 0.36	61	2.0
25	5.47 ± 0.24	120	6.25	16.2	3.09	5.42 ± 0.48	80	3.25
30	7.91 ± 0.30	252	9.0	21.2	2.36	5.93 ± 0.38	140	4.5
