# Supplementary material for: Rapid Brownian Motion Primes Ultrafast Reconstruction of Intrinsically Disordered Phe-Gly Repeats Inside the Nuclear Pore Complex
Source: Sci Rep. 2016 Jul 29;6:29991. doi: 10.1038/srep29991 (PMC4965864; doi:10.1038/srep29991)
Supplement: Supplementary Information [file srep29991-s1.pdf]

Rapid Brownian Motion Primes Ultrafast Reconstruction of Intrinsically Disordered Phe-Gly Repeats Inside the Nuclear Pore Complex

R. Moussavi-Baygi[1] and M. R. K. Mofrad[1,2*]

[1] Molecular Cell Biomechanics Laboratory, Departments of Bioengineering and Mechanical Engineering, University of California, Berkeley, CA, USA 94720
[2] Molecular Biophysics and Integrative Bioimaging Division, Lawrence Berkeley National Laboratory, Berkeley, CA, USA 94720

* Correspondence to: mofrad@berkeley.edu

Supporting Information

Brownian dynamics (1) used to simulate the 3D model of the yeast NPC central channel with eight spoke domains including all of the central FG-Nups. The 3D map of the NPC central channel (2), the upper and the lower half-spoke domains along with the tethering points of FG-Nups therein (3), and the sequence of the disordered domain of each yeast FG-Nup along with their anchor domains (4) are incorporated in our model.

The central channel is composed of eight identical spoke domains, composed of upper and lower half-spokes, and five FG-Nups per half-spoke (3). In this work, we first made these two half-spokes with all of known FG-Nups tethered to them (Fig. S1), and then the entire spoke domain was rotationally repeated eight times around the central axis of the pore to form the central channel. This procedure is consistent with the proposed assembly of the NPC *in vivo* (3, 5). The disordered domains of FG-Nups (4, 6) were extracted from the original sequences available in UniProt (see Table1). The anchor domains were taken from the work of Yamada *et al.* (4), while positions of tethering points on the spoke domain were calculated from the work of Alber *et al.* (3). The initial configurations of disordered domains were set straight, but cramped so that they could be fit within the diameter of the channel, consistent with our previous works (Fig. S1) (7, 8). Nonetheless, by running multiple independent initial conditions, we

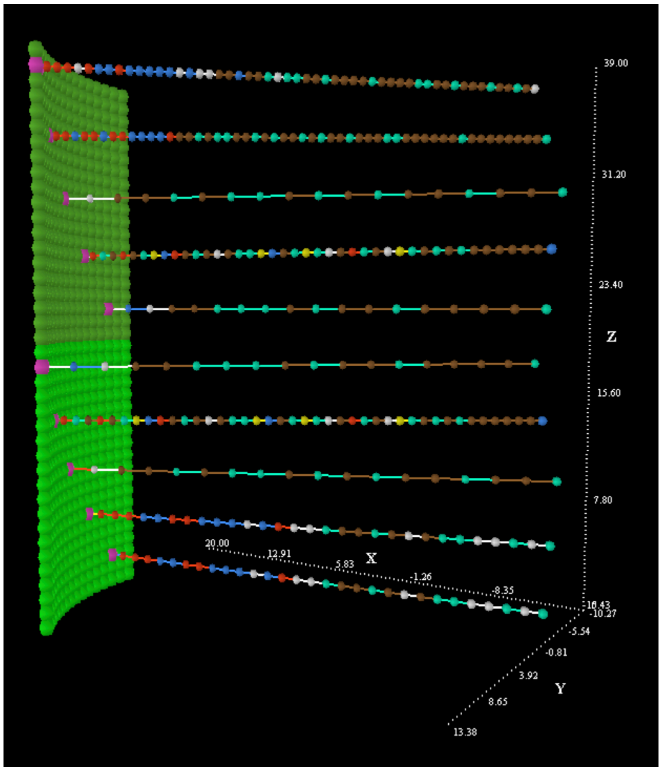

Figure S1 The single spoke with disordered FG-Nups being in their initial, cramped configurations. FG-Nups are modeled as bead-spring. Upper and lower spokes are colored in dark and light green, with the tethering point of each FG-Nup colored in pink. Each bead in the FG-Nup represents a monomer with the following coloring scheme: brown is hydrophobic (HB), yellow is hydrophobic-negative (HN), cyan is hydrophobic-positive (HP), red is purely negative (PN), blue is purely positive (PP), and white is hydrophilic with zero net charge (HL).

found that final conformations of FG-repeats are not sensitive to the initial configurations.

Eight spoke domains are modeled as rigid elements, while intrinsically disordered domains of FG-Nups are modeled as bead-spring elements (Fig. S1) using discrete wormlike chains (dWLC) with the persistence length of $l_p = 4.3\text{Å}$ (9). Each bead represents a monomer consisting of five successive amino acids (AAs), featuring the net charge of constituting AAs at physiological pH, hydrophobicity, and

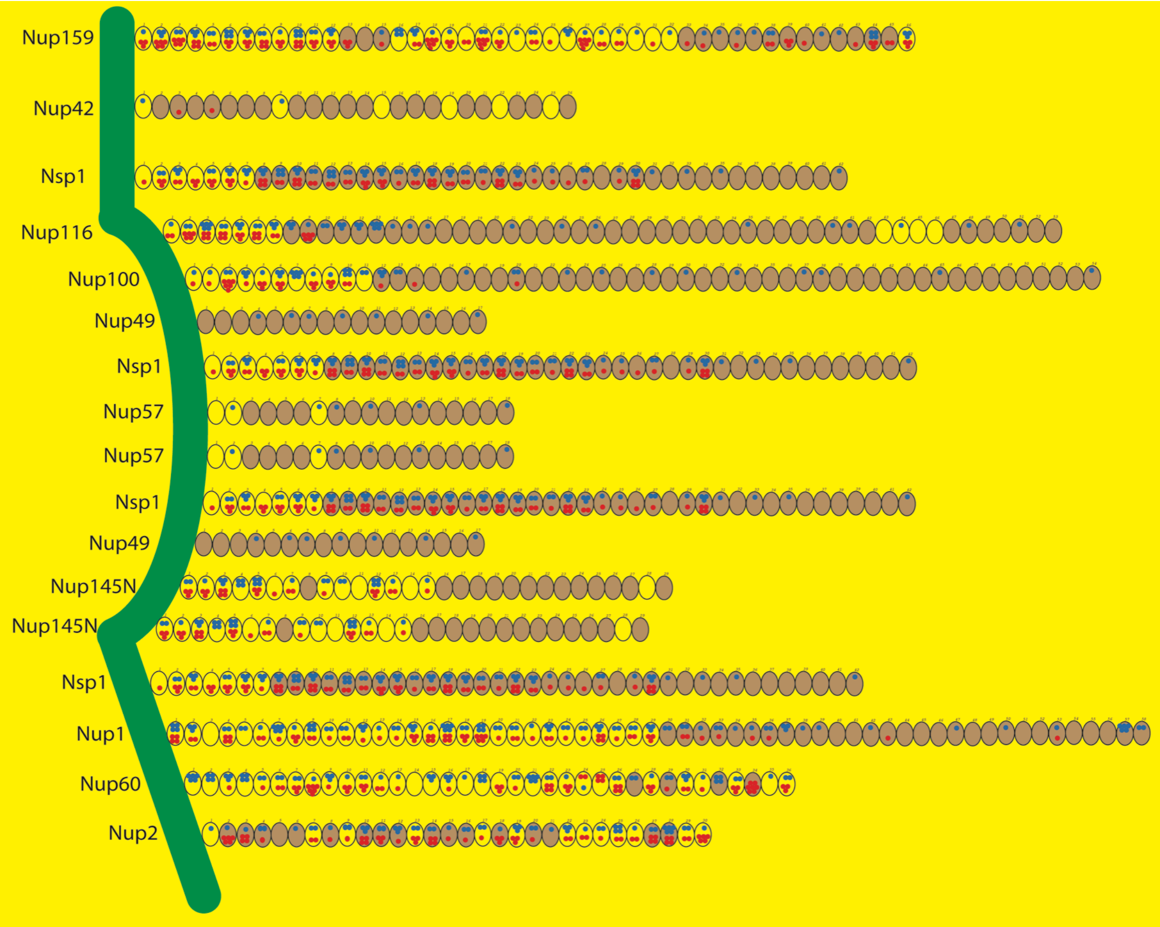

Figure S2. The 2D representation of an octagonal arm of the yeast NPC with a cytoplasmic filament, a spoke domain, and a nuclear basket rod. Each bead represents a monomer, which includes the positive and negative charges as well as the hydrophobicity of constituting residues. Positive and negative charges of a monomer are represented by adequate number of blue and red dots inside the oval. Hydrophobic monomers are tanned.

the total vdW volume (Fig. S2).

There are six different types of monomers considered in the current model: i) hydrophobic (HB), which have one or two Phe residues with the rest of residues being polar; ii) hydrophobic-negative (HN), which have both Phe residue and at least one negatively charged residue (Asp, Glu), iii) hydrophobic-positive

(HP), having Phe and at least one positively charged residue (Arg, Lys), iv) purely negative (PN), have at least one negatively charged residue without any hydrophobic residue, v) purely positive (PP) with at least one positively charge residue without any hydrophobic residue, and vi) hydrophilic (HL), which have only polar residues with the net zero charge.

The force-extension constitutive equation for dWLC is $F_{ij}^{wlc} = k_B T / l_p \left[0.25 \left(1 - r_{ij} / dL_c \right)^{-2} - 0.25 + r_{ij} / dL_c \right]$ (7, 8), where dL_c is the segmental contour length for a single monomer and equals the sum of lengths of the N- to C-termini of five successive AAs (10). The bending potential for dWLC is taken as $2U_{ijk}^{bend} = \left(\cos\left(\theta_{ijk} \right) - \cos\left(\theta_{ijk}^{0} \right) \right)^2$ with the small bending rigidity of $b = k_B T l_p / dL_c$ (7).

To imitate the polymers' self-avoiding property and excluded volume effects, a soft repulsion potential $U_{ij}^{rep} = \varepsilon \exp\left(-r_{ij} / \sigma \right)$ is used with $\varepsilon = 100.0 k_B T$ and $\sigma = 1.0 nm$ with the pairwise cutoff radii being the sum of vdW radii of two approaching monomers (11). The same potential is applied between monomers and the channel rigid wall to prevent penetration into the wall and represent the confinement.

The electrostatic interaction between two monomers with radii R_i and R_j is modeled using the linearized Poisson-Boltzmann equation (12), i.e. Deybe-Hückel approximation, with the central net charges (q_i, q_j) and the additive pairwise potential (13, 14): $U_{ij}^{elec} = q_i q_j \left[4\pi\varepsilon_0 \varepsilon_r (1 + \kappa R_i)(1 + \kappa R_j) r_{ij} \right]^{-1} \exp\left[-\kappa \left(r_{ij} - R_i - R_j \right) \right]$. Here ε_0 is the vacuum permittivity, ε_r the relative permittivity of the cytoplasmic environment that amounts to 45.0 (15), and $\kappa = \lambda^{-1}$ is the inverse Debye screening length, which is $1.0 nm$ for the cytoplasmic ionic strength (16). However, if the surface-to-surface distance of two monomers is less than λ, then the unscreened Coulombic potential is applied $U_{ij}^{Coul} = q_i q_j \left[4\pi\varepsilon_0 \varepsilon_r r_{ij} \right]^{-1}$ (17). The FG-repeat domains are mainly positively charged as opposed to NTRs that possess net negative charges (18).

Hydrophobic effects are relatively more subtle to model since they are mainly entropic in nature. In the current study, we tested a wide range of hydrophobic force fields (19-24). To determine the optimized force filed for FG-Nups five criteria were implemented: *i*) availability of the force field parameters for FG-Nups, *ii*) ability to represent the overall known physiological properties of FG-Nups, *iii*) minimal number of parameters involved, *iv*) numerical efficiency, and *v*) simplicity of implementation in the simulation. Remarkably, we found the double-parameter exponential potential energy was the most optimized force field for FG-Nups. This potential is proposed for purely hydrophobic interaction energy between two hydrophobic surfaces in a polar solution like water (25): $U_{ij}^{hphb} = -2\gamma \exp\left(-r_{ij} / \mu \right)$ with $\gamma = 0.4\ k_B T / nm^2$ representing the hydrophobic affinity per unit area and $\mu = 1.0 nm$ being the characteristic length of interaction.

In this work, we take into account the hydrophobic interaction between all Phe residues—the most abundant hydrophobic residue within the disorder domains of FG-Nups. Thus, the area of interaction is

chosen as the solvent-accessible surface area (SASA) of the nonpolar side chain of Phe in its extended conformation, i.e. $175\ \mathring{A}^2$ (11).

The main parameters used in potential energies as described above are the same as those used in previous 2D model. For example, the persistence length of discretized wormlike chains, the repulsion potential energy, and the hydrophobic interaction potential energy could be borrowed from our 2D model (7, 8).

The entire systems is placed in an aqueous bath with cytoplasmic viscosity and ionic strength at physiological pH = 7.3 and the body temperature of 37 C. The integrated form of the coupled Langevin equations of motion are then solved explicitly in time for all monomers: $\mathbf{r}_i(t+\Delta t)=\mathbf{r}_i(t)+\left(D_i/k_BT\right)\mathbf{F}_i(t)\Delta t+\mathbf{\Lambda}_i$, where D_i is the diffusion coefficient for monomer i and is related to the friction coefficient ζ_i through the Einstein–Smoluchowski relation: $D_i = k_BT/\zeta_i$. The friction coefficient is obtained from the Stokes' relation $\zeta_i = 6\pi\eta R_i^{hyd}$ with $\eta = 5\,\mathrm{cP}$ being the cytoplasmic viscosity and R_i^{hyd} the hydrodynamic radius of the monomer (26). In addition, $\mathbf{\Lambda}_i$ is the random displacement vector that represents the random motion of the monomer in implicit solvent and follows a Gaussian distribution with a zero mean and the variance of $2D_i\Delta t$.

Cargos are also modeled as rigid objects with desired dimensions and shapes, globular or elongated. The repulsion, electrostatic, and hydrophobic potentials are applied between the cargo surface and monomers/wall in the same manner outlined above for monomer-monomer and monomer-wall interactions. To represent NTRs bound to the cargo adequate number of hydrophobic binding spots are placed on the cargo surface (see Table 1). The hydrophobic binding spots are restricted to only one side of the cargo surface, consistent with the localization of the hydrophobic binding patches on the convex surface of the kap β (27). To model the NTR-FG hydrophobic interactions, the same attractive interaction potential like the one used to model hydrophobic Phe-Phe interaction, $U_{ij}^{hphb} = -2\gamma\exp\left(-r_{ij}/\mu\right)$, is used. However, the NTR-Phe hydrophobic affinity is set stronger than Phe-Phe affinity (6) to yield the nanomolar apparent dissociation constant between NTRs and FG-Nups (27) through $k_d = \exp\left(U_{NTR-F}^{hphb}/k_BT\right)$. This yields a hydrophobic affinity density between a NTR binding spot and a FG-motif of $\gamma_{NTR-Phe} = 2.0\ k_BT/nm^2$. The characteristic length of hydrophobic interaction between NTR binding spot and FG, however, remains the same as that for Phe-Phe hydrophobic interaction and is $\mu = 1.0\,nm$. Furthermore, the area of interaction is chosen as the solvent-accessible surface area (SASA) of the nonpolar side chain of Phe in its extended conformation, i.e. $175\ \mathring{A}^2$ (11). Moreover, the net number of negative charges on the NTRs' surfaces is taken as -30, consistent with the experimental observations (18).

The diffusion coefficient of the globular cargo is computed from the Einstein-Smoluchowski relation above. For the elongated cargo the diffusion coefficient has perpendicular and parallel components (28): $D_{cyl} = (1/3)\left(2D_{cyl}^{\perp}+D_{cyl}^{\parallel}\right)$, where $D_{cyl}^{\parallel} = \left(k_BT/2\pi\eta h\right)\left(\ln p - 0.207 + 0.980p^{-1} - 0.133p^{-2}\right)$ and $D_{cyl}^{\perp} = \left(k_BT/4\pi\eta h\right)\left(\ln p + 0.839 + 0.185p^{-1} + 0.233p^{-2}\right)$ with $p = h_{cyl}/2R_{cyl}$. This formulation has

been successfully applied to elongated macromolecules in the context of Brownian dynamics (29). The hydrophobic side of the cargo is faced toward the FG-repeats upon entering the central channel.

To get statistically reliable results, independent simulations are launched with different initial conditions, meaning, the initial random seeds for each simulation is different.

Throughout the simulations, all physical parameters are dimensionless with respect to energy, time, and length with the unit values of $1.0 k_B T$, $0.1 ns$, and $1.0 nm$ respectively. After an equilibration of 10 *μs*, the simulation started and continued for 20 *ms*, long enough to span the entire nucleocytoplasmic transport process, and presumably allowing for the disordered domains to assume all possible conformations that can be adopted in the absence of any cargo.

Discretization scheme

To ensure our results are not dependent on spatial resolution of the bead-spring model (the level of discretization), a sensitivity analysis was performed. Specifically, a coarser discretization level, i.e. 15

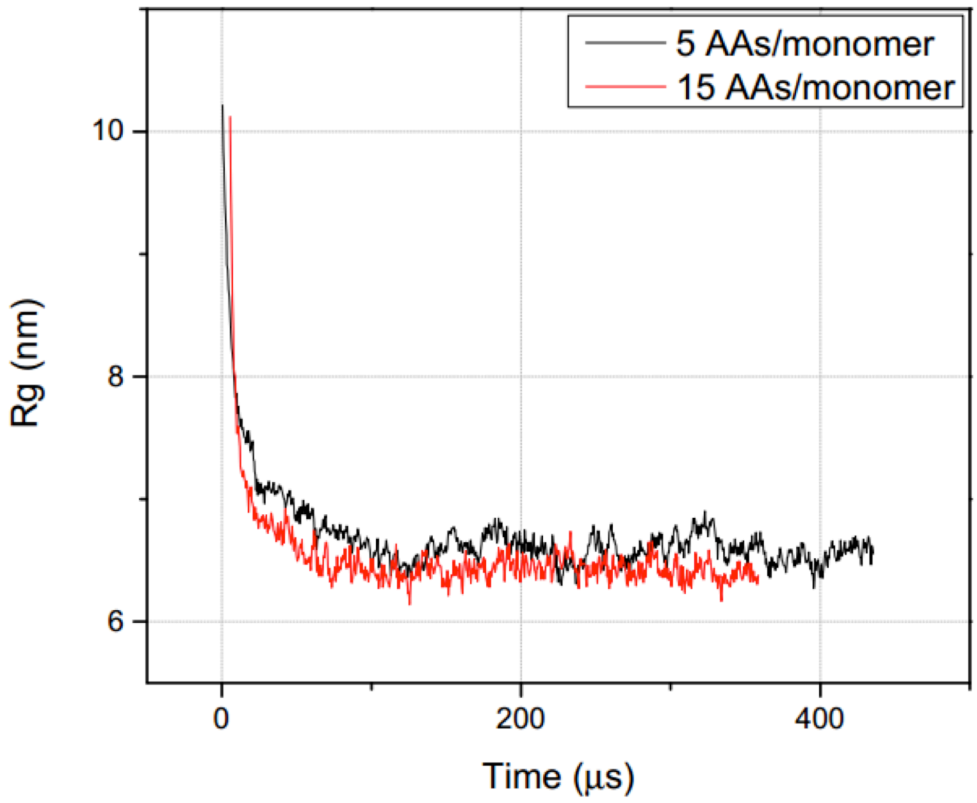

Figure S3. The time-evolution of the radius of gyration of FG-Nups in two different discretization schemes: five AAs per monomer, and 15 AAs per monomer.

AAs/monomer, was performed and the time evolution of the averaged radii of gyration of FG-repeats, R_g, was examined over $400\mu s$. It appears that the time evolution as well as the average values of R_g exhibit similar patterns in both of the discretization levels (Fig. S3), and thus the model is not sensitive to the discretization level, suggesting that the spatial resolution does not bias the conformational behavior of FG-repeats. It must be noted that the discretization cannot go too fine as the model should be coarse enough to assure that the assumption of eliminating atomistic hydrogen bonds between solvent-solvent, solvent-reside, and residue-residue is valid. We thus adhere to a five-AAs/monomer coarse-graining level throughout the presented simulations, which is coarse enough to safely ignore the explicit hydrogen bonds, treating them with the stochastic Langevin treatment, and is fine enough to show the dynamics and distribution of hydrophobic and charged residues.

Pre-exposed hydrophobic and charged monomers of cramped FG-repeats in the confined geometry of the central channel

The lengths of FG-repeats localized to the NPC central channel are in the range of 85 to 272 nm (Table 1) with an average length of 180 nm. Thus, the initial configurations of FG-repeats in our simulations are cramped so that they can be fit in the 40-nm wide central channel, leading to hydrophobic as well as the charged monomers being pre-exposed.

We thus asked whether the formation of the observed dense FG-meshwork inside the central channel was potentially an artifact caused by the pre-exposed hydrophobic as well as charged residues from the same or different FG-Nups due to their initially cramped configurations within the channel. To address this question, we removed the initial pre-exposing by artificially enlarging the channel diameter, and thus, relaxing the crampedness while keeping the positions of tethering points unaltered. The artificial channel had the same height as the central channel (40 *nm*) but its diameter is $550\,nm$ (Fig. S4-A), a bit more than twice the longest disordered FG-Nup, i.e. Nup 100 (4). The initial configurations of disordered FG-Nups were set to their fully extended lengths, consistent with previous molecular dynamics studies performed in an open (unconfined) geometry (30, 31), ensuring that hydrophobic as well as charged residues along the same or different FG-Nups were not initially pre-exposed (Fig. S4-A).

FG-Nups within the artificial channel were first equilibrated for 5 μs (Fig. S4-B), during which they rapidly shrunk and deposited on the channel wall. After that, the channel diameter was shrunk in a stepwise manner to reach its native dimension, i.e. $40\ nm$. In each step, FG-Nups inside the new artificial channel were equilibrated for another 5 μs (Fig. S4-C to G). Finally, after the diameter was downsized to $40\ nm$ the FG-meshwork formed and occupied the channel with similar average density and conformations as those in the native channel. This proved that formation of the FG-meshwork inside the native central channel in our model is not an artifact of the pre-exposed hydrophobic or charged

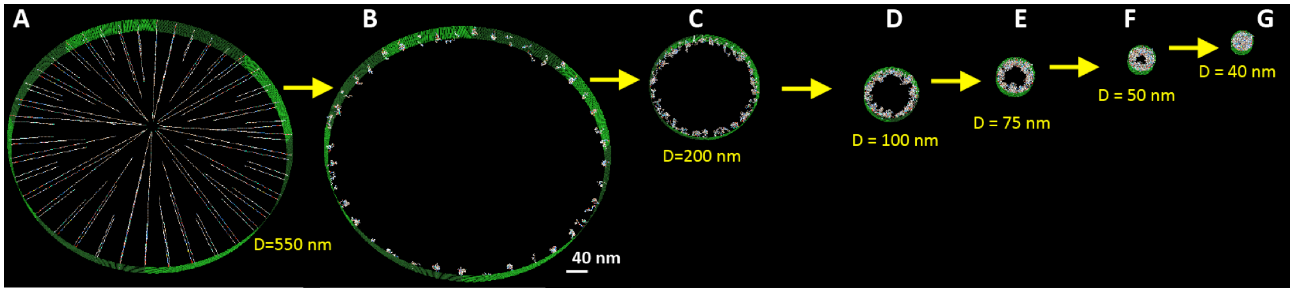

Figure S4. Starting from an artificially large channel, the channel diameter is decreased stepwise, and FG-Nups are equilibrated in each step. A) The initial configuration of the artificial central channel with unstructured domains of FG-Nups being in their fully extended length configuration. The diameter of the artificial channel is 550 nm, which guarantees monomers do not meet nor the unstructured domains cross each other. The height of the artificial channel is kept the same as that for the original yNPC central channel. B) The artificial channel after equilibration.
C-G) The diameter of the artificial channel is shrunk stepwise to the final value of 40 nm. In each step, the system is equilibrated for 5 µs before shrinking the diameter to the next step. In the final step, the channel-filling meshwork is reached, as in the original NPC channel.

residues.

The average configurational entropic cost sets the theoretical minimum number of hydrophobic binding spots: larger macromolecules need more NTR to overcome permeability barrier.

When a large active cargo enters the central channel, the disordered FG-repeats in its vicinity experience a reduction in their configurational entropy to locally accommodate the cargo. The decrease in configurational entropy of a single monomer, Δs_{mon}, can be estimated by calculating the reduction in the available space to the monomer (32) as:

$$\Delta s_{mon} = k_B \ln\left(\frac{V_0 - V_c}{V_0}\right), \qquad (2)$$

where V_c is the volume of the active cargo, and V_0 the available space to the monomer before the cargo enters the channel, taking into account the excluded volume effects of other monomers: $V_0 = V_{chn} - (N-1)v_{mon}$. Here, V_{chn} is the bare channel volume, N the total number of monomers, and v_{mon} the vdW volume of a single monomer (11).

If ρ is the average number density of the monomers inside the channel, for a single active cargo, on average, $n_{mon} = \rho V_c$ monomers must be dislocated to locally accommodate the cargo entirely inside the FG-meshwork, leading to an entropic cost of $\Delta S = n_{mon}\Delta s_{mon}$. This is, of course, a minimal estimation for the entopic cost because we only consider dislocation of monomers immediate to the cargo surface and ignore farther layers.

The configurational entropic cost is compensated by *entropically* and *energetically* favorable hydrophobic, and to a lesser extent, *energetically* favorable electrostatic interactions between NTR and FG-repeats. Thus, ΔS for a given cargo can be used to determine the minimal number of hydrophobic patches on the active cargo surface required to overcome the lower bound of the configurational entropic barrier. For example, for a globular cargo of 15 *nm* in diameter, $\Delta s_{mon} \cong -0.04k_B$ and $n_{mon} \cong 300$, and thus the lower bound of the entropic cost is $\Delta S \cong -12k_B$. Therefore, at the cell temperature the free energy gain required to overcome this entropic barrier must be greater than $12k_BT$. Considering the NTR-FG-repeats hydrophobic interaction, at least six hydrophobic patches would be required on the surface of a 15-*nm* cargo for the energy gain of $12k_BT$. However, in reality this number of hydrophobic patches is not sufficient for this cargo to overcome the entropic barrier and perforate the FG-meshwork, at least during the simulation times in the current study. Yet, this calculation can set a theoretical lower bound to estimate the minimal number of hydrophobic binding spots required for a cargo with a certain size to overcome the entropic barrier of the NPC. It should be remarked, however, that this is a rough estimation; for simplicity we used an average value of the density for the monomers inside the channel and also we ignored the end-tethering of the FG-repeats, which otherwise would not be straightforward to find the entropic cost. Moreover, the solvent translation entropy was ignored in our implicit solvent model. Nevertheless, this theoretical approach shows that larger cargos need more number of hydrophobic binding spots, and thus more NTRs, to overcome the permeability barrier, as was observed experimentally (33).

Table S1. Summary of intrinsically disordered domains of the yeast FG-Nups adopted in the current study

FG-Nup	UniProt accession number	Approximate localization region	# per octagonal arm	# per upper spoke	# per lower spoke	# of AAs in disordered domain*	# of FG-motifs in disordered domain*	# of Phe in disordered domain*	# of Gly in disordered domain*	Net charge in disordered domain*
Nup159	P40477	Cytoplasmic side	1	–	–	685	18	45	55	-38
Nup42	P49686	Cytoplasmic side	1	–	–	382	21	35	54	11
Nsp1	P14907	Cytoplasmic, central, and nuclear sides	4	1	1	617	28	57	62	8
Nup116	Q02630	Central channel	1	1	0	789	27	50	126	11
Nup100	Q02629	Central channel	1	1	0	800	34	54	111	12
Nup49	Q02199	Central channel	2	1	1	251	12	18	53	7
Nup57	P48837	Central channel	2	1	1	255	14	19	59	7
Nup145	P49687	Central channel	2	0	2	433	9	24	43	8
Nup1	P20676	Nuclear side	1	–	–	857	15	64	76	19
Nup60	P39705	Nuclear side	1	–	–	151	4	23	20	13
Nup2	P32499	Nuclear side	1	–	–	441	10	34	20	-8

*The disordered domains are taken from Yamada et al. (4).

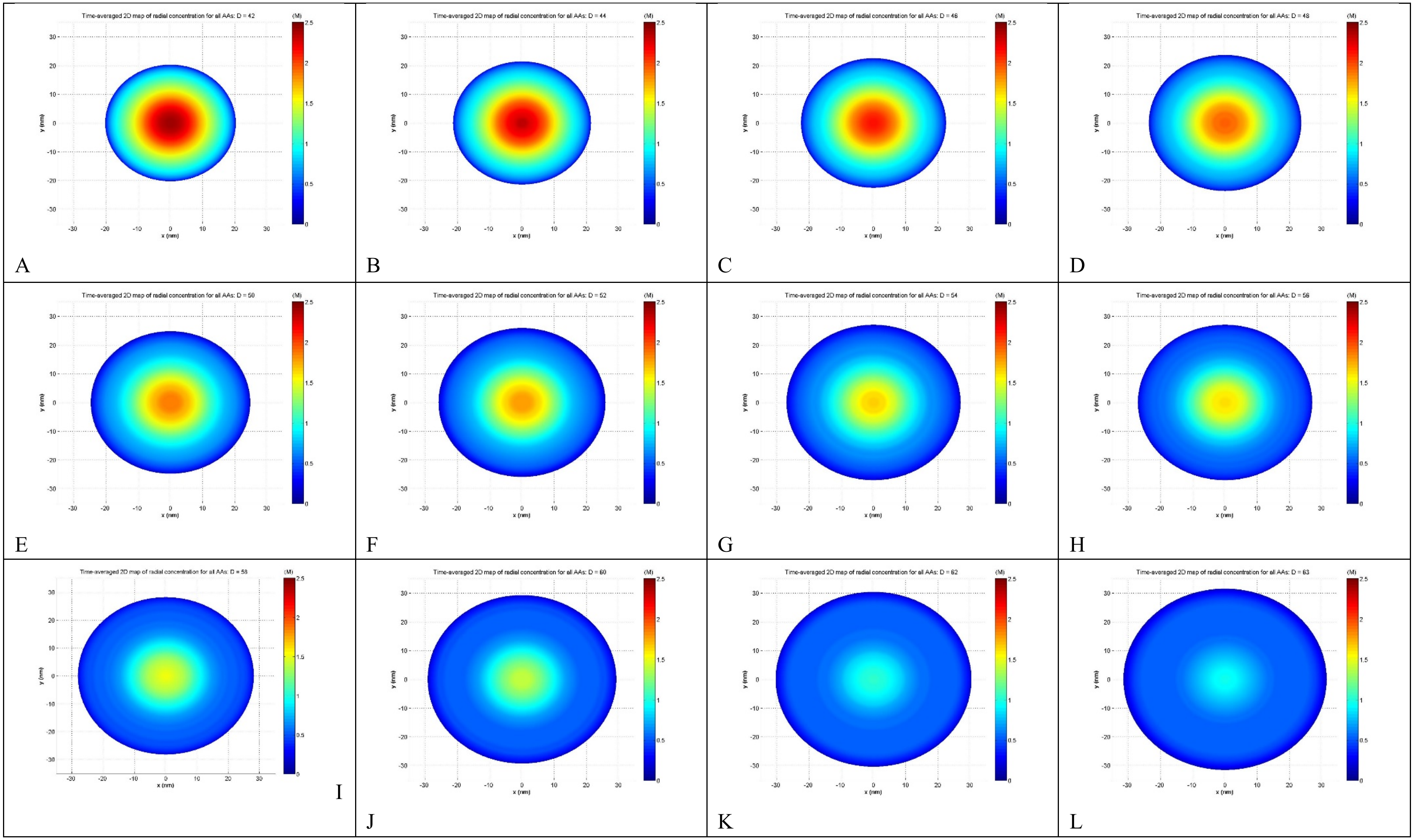

Figure S5. The dependence of the intra-FG-meshwork zone formation on the diameter of the channel. The channel diameter is increased from 42 nm in panel A to 64 nm in panel L. From each panel to the next, the increment in the channel diameter is 2 nm. Colorbars in all panels have the same limits (0 to 2.5 M), and x and y axes‘ limits are -30 to 30 nm.

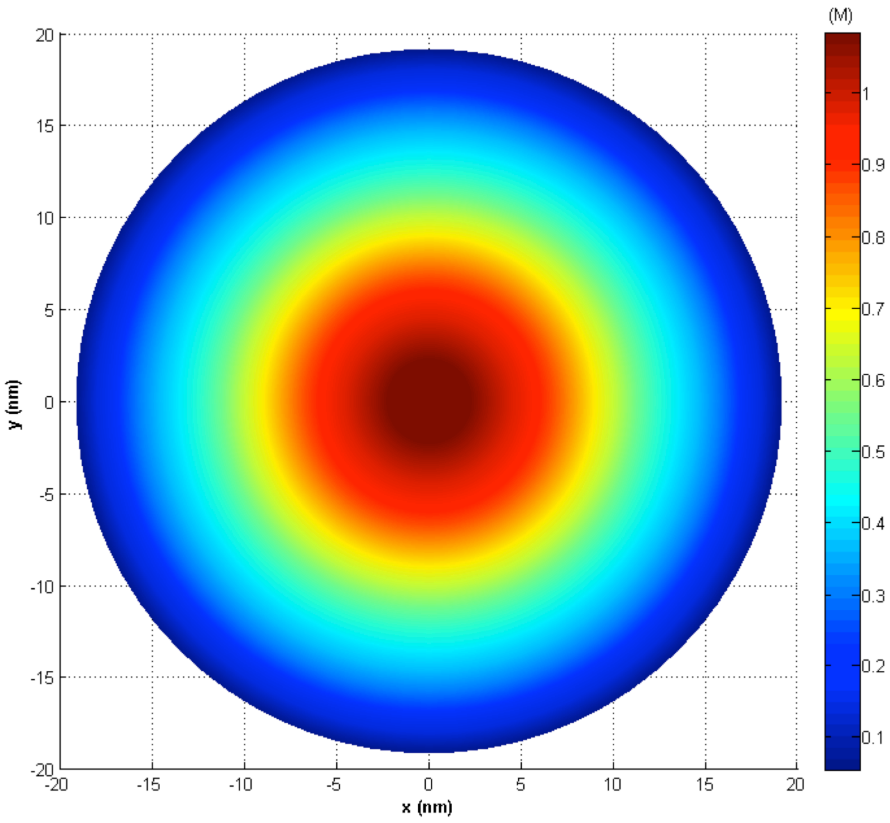

Figure S6. Time-averaged 2D map of radial concentration of all Phe residues within the FG-repeat domains. The concentrations are averaged along the channel main axis.

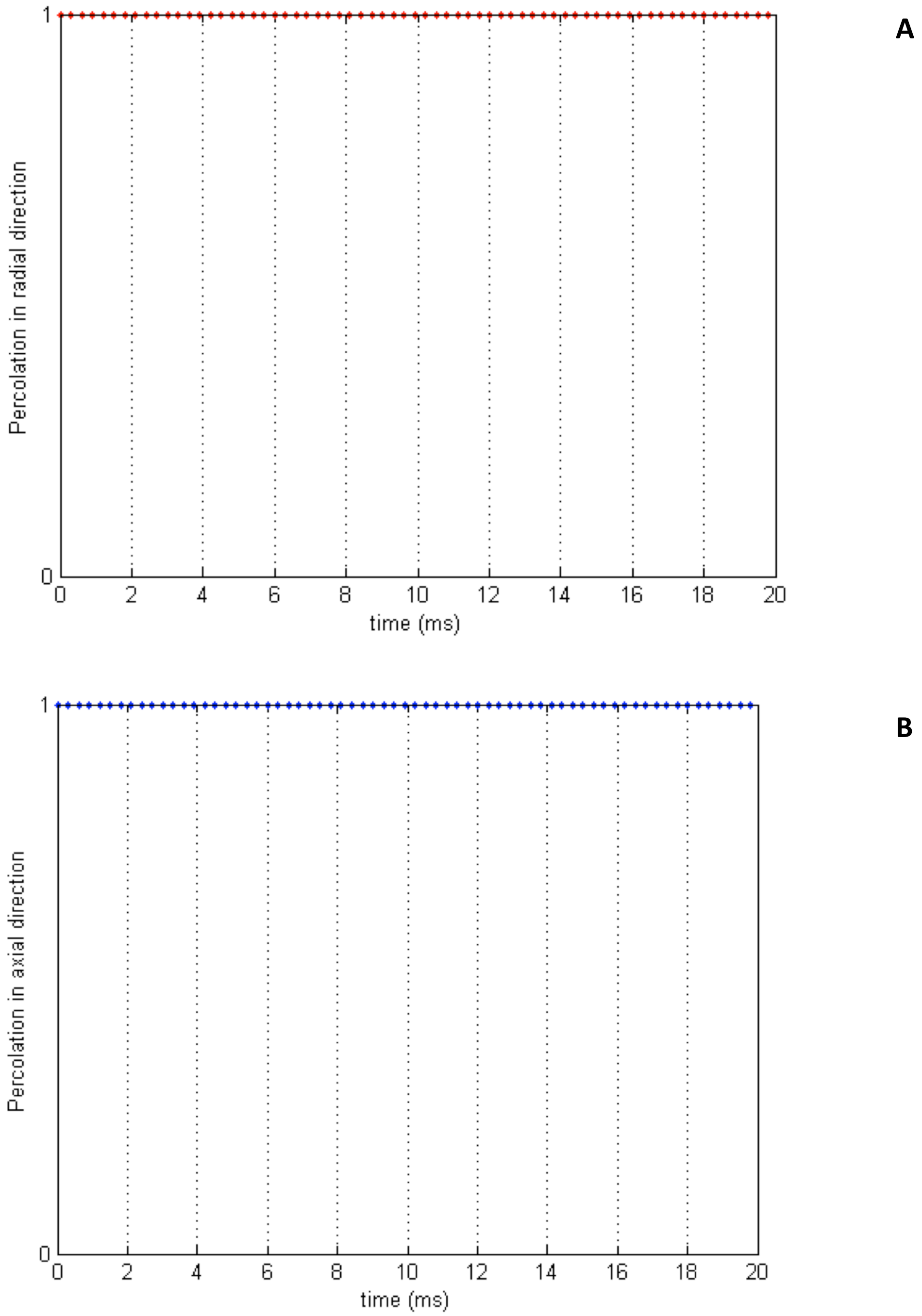

Figure S7 Percolation of the FG-meshwork in the radial and the axial directions within the central channel during the simulation. Vertical axis shows whether percolation occurs (=1) or not (=0) for a particular configuration corresponding to a certain simulation time. Adding up those numbers, we can calculate the probability of percolation for the total simulation time of 20 ms. The probability of percolation is 100% in radial as well as the axial directions, meaning the FG-meshwork always forms a connected pathway in both directions. A) Radial direction, and B) axial direction.

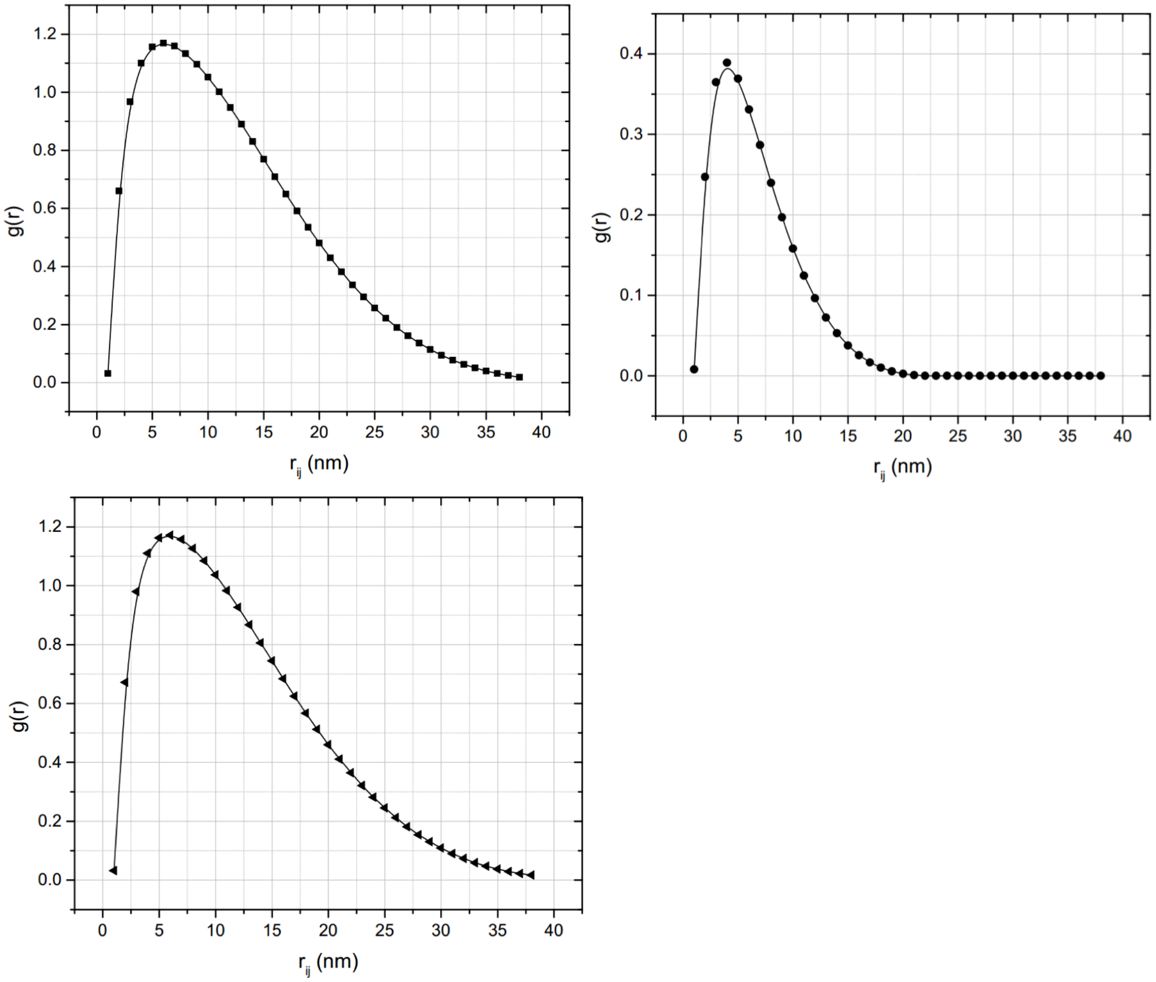

Figure S8. The pair distribution function (PDF) is used to calculate the frequencies and distances of particles' pairwise separations inside a fluid with a given structure and density relative to a fluid with a completely random structure at the same density. Thus, it can provide profound insights into the microstructure of the FG-meshwork in the current study. The monomer-monomer PDF plot of the FG-meshwork for A) the entire meshwork (rectangles), B) high-density zone in the middle (circles), and C) low-density zone near the wall (triangles).

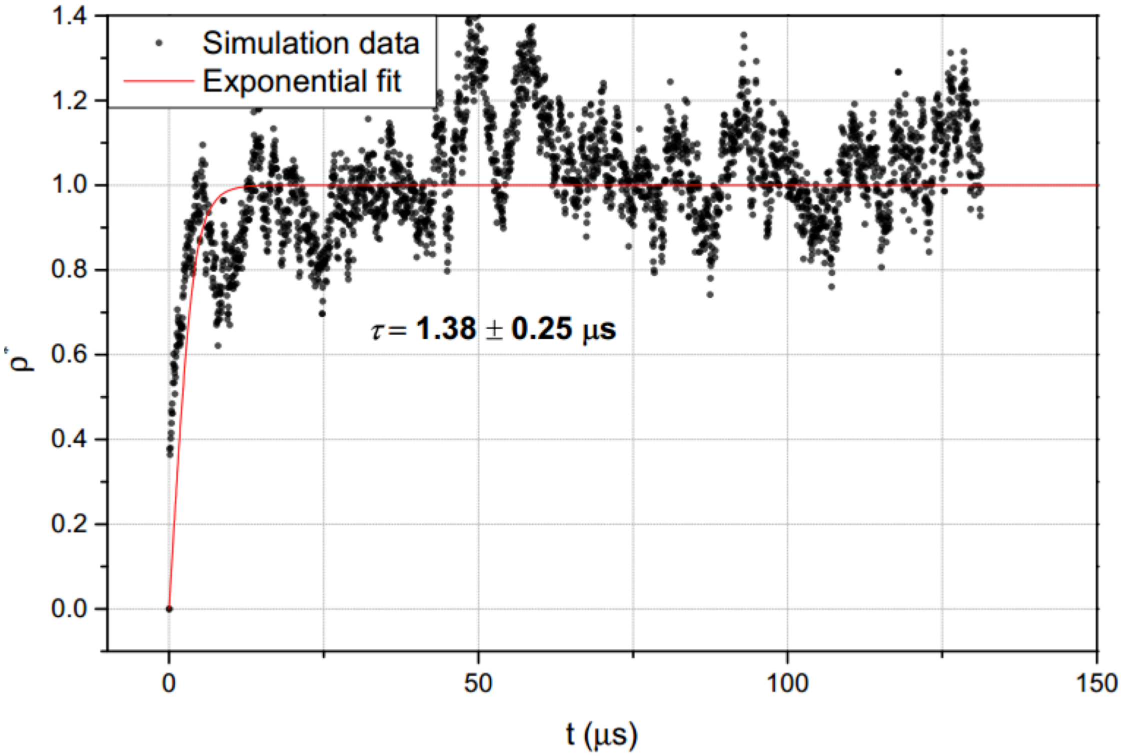

Figure S9. The reconstruction patter of the non-FG-meshwork, where all of the Phe residues are replaced by polar residues with the same vdW dimension. The cargo in this case is a 20-nm NTR-bearing globular macromolecule.

Movies' legends

Movie S1: Concentration fluctuations of unstructured domains in the central channel.

Movie S2: Side-view of the time-dependent, 3D spatial density map of hydrophobic interactions inside the central channel

Movie S3: Top-view of the time-dependent, 3D spatial density map of hydrophobic interactions inside the central channel

Movie S4: Side-view of the steered nucleocytoplasmic transport of the NTR-bearing spherical cargo across the central channel

Movie S5: Top-view of the steered nucleocytoplasmic transport of the NTR-bearing spherical cargo across the central channel

Movie S6: Side-view of the steered nucleocytoplasmic transport of the NTR-bearing elongated cargo across the central channel

Movie S7: Top-view of the steered nucleocytoplasmic transport of the NTR-bearing elongated cargo across the central channel

Movie S8: The globular nonspecific cargo cannot penetrate into the FG-meshwork with nudging forces up to 70 pN

References

1. Ermak DL & Mccammon JA (1978) Brownian Dynamics with Hydrodynamic Interactions. *J Chem Phys* 69(4):1352-1360
2. Yang Q, Rout MP, & Akey CW (1998) Three-Dimensional Architecture of the Isolated Yeast Nuclear Pore Complex: Functional and Evolutionary Implications. *Molecular Cell* 1:223–234
3. Alber F*, et al.* (2007) The molecular architecture of the nuclear pore complex. *Nature* 450(7170):695-701
4. Yamada J*, et al.* (2010) A bimodal distribution of two distinct categories of intrinsically-disordered structures with separate functions in FG nucleoporins. *Mol Cell Proteomics*
5. Alber F*, et al.* (2007) Determining the architectures of macromolecular assemblies. *Nature* 450(7170):683-694
6. Patel SS, Belmont BJ, Sante JM, & Rexach MF (2007) Natively unfolded nucleoporins gate protein diffusion across the nuclear pore complex. *Cell* 129(1):83-96
7. Moussavi-Baygi R, Jamali Y, Karimi R, & Mofrad MR (2011) Brownian dynamics simulation of nucleocytoplasmic transport: a coarse-grained model for the functional state of the nuclear pore complex. *PLoS Comput Biol* 7(6):e1002049
8. Moussavi-Baygi R, Jamali Y, Karimi R, & Mofrad MR (2011) Biophysical coarse-grained modeling provides insights into transport through the nuclear pore complex. *Biophys J* 100(6):1410-1419
9. Lim RY*, et al.* (2006) Flexible phenylalanine-glycine nucleoporins as entropic barriers to nucleocytoplasmic transport. *P Natl Acad Sci USA* 103(25):9512-9517
10. Ohashi T, Galiacy SD, Briscoe G, & Erickson HP (2007) An experimental study of GFP-based FRET, with application to intrinsically unstructured proteins. *Protein science : a publication of the Protein Society* 16(7):1429-1438
11. Creighton TE (1993) *Proteins : structures and molecular properties* (W.H. Freeman, New York) 2nd Ed pp xiii, 507 p.
12. Honig B & Nicholls A (1995) Classical electrostatics in biology and chemistry. *Science* 268(5214):1144-1149
13. Dlugosz M & Trylska J (2011) Diffusion in crowded biological environments: applications of Brownian dynamics. *BMC Biophys* 4:3
14. Medina-Noyola M & McQuarrie DA (1980) On the interaction of spherical double layers. *The Journal of chemical physics* 73(12):6279-6283
15. Fikus M & Pawlowski P (1989) Bioelectrorheological model of the cell. 2. Analysis of creep and its experimental verification. *Journal of theoretical biology* 137(4):365-373
16. Nelson P (2013) *Biological Physics* (Freeman, New York).
17. Phillips R, Kondev J, Theriot J, & Garcia H (2012) *Physical biology of the cell* (Garland Science).
18. Colwell LJ, Brenner MP, & Ribbeck K (2010) Charge as a Selection Criterion for Translocation through the Nuclear Pore Complex. *PLoS Comput Biol* 6(4):-
19. Claesson PM & Christenson HK (1988) Very long range attractive forces between uncharged hydrocarbon and fluorocarbon surfaces in water. *The Journal of Physical Chemistry* 92(6):1650-1655
20. Yoon R-H, Flinn DH, & Rabinovich YI (1997) Hydrophobic interactions between dissimilar surfaces. *Journal of colloid and interface science* 185(2):363-370
21. Donaldson SH, Lee CT, Chmelka BF, & Israelachvili JN (2011) General hydrophobic interaction potential for surfactant/lipid bilayers from direct force measurements between light-modulated bilayers. *Proceedings of the National Academy of Sciences* 108(38):15699-15704

22. Curtis R, Steinbrecher C, Heinemann M, Blanch H, & Prausnitz J (2002) Hydrophobic forces between protein molecules in aqueous solutions of concentrated electrolyte. *Biophysical chemistry* 98(3):249-265
23. Hummer G (1999) Hydrophobic force field as a molecular alternative to surface-area models. *Journal of the American Chemical Society* 121(26):6299-6305
24. Kellogg GE, Semus SF, & Abraham DJ (1991) HINT: a new method of empirical hydrophobic field calculation for CoMFA. *Journal of computer-aided molecular design* 5(6):545-552
25. Israelachvili JN (1992) *Intermolecular and surface forces* (Academic Press, London) 2nd Ed.
26. Fischer H, Polikarpov I, & Craievich AF (2004) Average protein density is a molecular-weight-dependent function. *Protein Sci* 13(10):2825-2828
27. Peters R (2009) Translocation through the nuclear pore: Kaps pave the way. *Bioessays* 31(4):466-477
28. Tirado MM & de la Torre JG (1979) Translational friction coefficients of rigid, symmetric top macromolecules. Application to circular cylinders. *The Journal of chemical physics* 71(6):2581-2587
29. Klenin K, Merlitz H, & Langowski J (1998) A Brownian dynamics program for the simulation of linear and circular DNA and other wormlike chain polyelectrolytes. *Biophysical Journal* 74(2):780-788
30. Miao L & Schulten K (2009) Transport-related structures and processes of the nuclear pore complex studied through molecular dynamics. *Structure* 17(3):449-459
31. Miao L & Schulten K (2010) Probing a structural model of the nuclear pore complex channel through molecular dynamics. *Biophys J* 98(8):1658-1667
32. Teraoka I (2002) *POLYMER SOLUTIONS* (John Wiley & Sons, Inc., New York).
33. Ribbeck K & Gorlich D (2002) The permeability barrier of nuclear pore complexes appears to operate via hydrophobic exclusion. *EMBO J* 21(11):2664-2671
